# Orientation of the Calcium Channel β Relative to the α_1_2.2 Subunit Is Critical for Its Regulation of Channel Activity

**DOI:** 10.1371/journal.pone.0003560

**Published:** 2008-10-29

**Authors:** Iuliia Vitko, Aleksandr Shcheglovitov, Joel P. Baumgart, Imilla I. Arias-Olguín, Janet Murbartián, Juan Manuel Arias, Edward Perez-Reyes

**Affiliations:** Department of Pharmacology and Neuroscience Graduate Program, University of Virginia, Charlottesville, Virginia, United States of America; University of Cincinnati, United States of America

## Abstract

**Background:**

The Ca_v_β subunits of high voltage-activated Ca^2+^ channels control the trafficking and biophysical properties of the α_1_ subunit. The Ca_v_β-α_1_ interaction site has been mapped by crystallographic studies. Nevertheless, how this interaction leads to channel regulation has not been determined. One hypothesis is that βs regulate channel gating by modulating movements of IS6. A key requirement for this direct-coupling model is that the linker connecting IS6 to the α-interaction domain (AID) be a rigid structure.

**Methodology/Principal Findings:**

The present study tests this hypothesis by altering the flexibility and orientation of this region in α_1_2.2, then testing for Ca_v_β regulation using whole cell patch clamp electrophysiology. Flexibility was induced by replacement of the middle six amino acids of the IS6-AID linker with glycine (PG6). This mutation abolished β2a and β3 subunits ability to shift the voltage dependence of activation and inactivation, and the ability of β2a to produce non-inactivating currents. Orientation of Ca_v_β with respect to α_1_2.2 was altered by deletion of 1, 2, or 3 amino acids from the IS6-AID linker (Bdel1, Bdel2, Bdel3, respectively). Again, the ability of Ca_v_β subunits to regulate these biophysical properties were totally abolished in the Bdel1 and Bdel3 mutants. Functional regulation by Ca_v_β subunits was rescued in the Bdel2 mutant, indicating that this part of the linker forms β-sheet. The orientation of β with respect to α was confirmed by the bimolecular fluorescence complementation assay.

**Conclusions/Significance:**

These results show that the orientation of the Ca_v_β subunit relative to the α_1_2.2 subunit is critical, and suggests additional points of contact between these subunits are required for Ca_v_β to regulate channel activity.

## Introduction

Calcium influx via voltage-gated Ca^2+^ channels (Ca_v_) play vital roles in cell physiology, such as triggering muscle contraction and hormone secretion [1]. Both the amount of Ca^2+^ that enters a cell, and where in the cell it enters, are highly regulated. To fulfill these specialized roles, Ca^2+^ channels have evolved into multimeric complexes composed of an α_1_, α_2_δ, and β, and each of these subunits has evolved such that there are ten α_1_ genes, four α_2_δ genes, and four β genes. Other mechanisms by which cells can fine tune Ca^2+^ channel activity include: alternative splicing of these Ca_v_ genes, regulation by calmodulin and G protein βγ subunits, and phosphorylation by protein kinases. One of the first findings from studies with recombinant Ca_v_ channels was the dominant role of Ca_v_β subunits [2]–[4]. Although the α1 subunit contains the channel pore, the voltage sensors, and most of the drug binding sites, the auxiliary subunits regulate all of these structures to increase channel opening, shift the voltage and time dependence of channel gating, and to increase drug affinity [5], [6].

Ca_v_β subunits are known to bind with high affinity to the I–II loop of HVA α1 subunits [7]. This site has been termed the alpha-interacting domain (AID), and is located 22 amino acids (a.a.) away from the C-terminus of the last transmembrane segment of repeat I (IS6). Recently three groups reported the crystal structure of Ca_v_β, either alone or in complex with a synthetic peptide corresponding to the AID [8]–[10]. These results confirmed the hypothesis that Ca_v_β subunits were part of the MAGUK protein family [11], and showed how the α-helical AID is embedded in the guanylate kinase (GK) domain of Ca_v_β. Despite such a clear picture of where it binds to α1, it is unclear how this translates into channel regulation. In fact, splice variants of Ca_v_β have been found that lack the GK domain, yet are still able to regulate the probability of channel opening, P_o_ [12], [13].

Previously we have shown that some aspects of Ca_v_β regulation could be conferred on a T-type channel α1 subunit (α_1_3.1) by transfer of the AID region from α_1_2.2 [14]. Similar to their regulation of HVA channels, Ca_v_β shifted the voltage dependence of activation to more hyperpolarized potentials, and increased the amount of current observed at the end of a sustained pulse. These studies provided the first evidence that β regulation required a rigid linker between IS6 and the AID, thereby providing support for the direct coupling hypothesis [15], which postulates that Ca_v_β alters movements of the IS6 segment that occur during gating. Notably missing from the α_1_3.1-2.2 chimera was Ca_v_β's regulation of channel P_o_ and closed state inactivation, which has been observed with wild-type N-type channels [16], [17]. Due to these limitations, we have now tested the direct coupling hypothesis by mutating α_1_2.2 directly. We show that deletion of a single amino acid in the IS6-AID linker is sufficient to abolish most aspects of Ca_v_β regulation (except trafficking to the plasma membrane). This result seemingly contradicts the direct-coupling hypothesis, and highlights the importance of β's orientation with respect to α_1_ in allowing interaction with its gating machinery.

## Results

**Figure 1:**
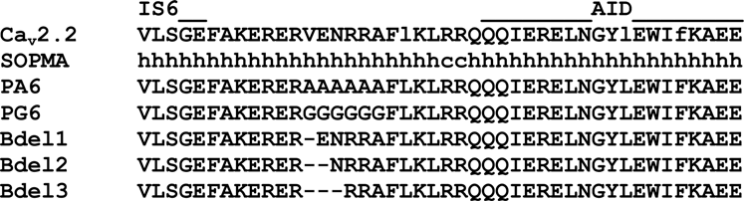
Altering the structure of the linker between AID and IS6. Amino acid sequence of the amino-terminal portion of the I–II loop of α_1_2.2, and location of the following mutations: poly-glycine (PG6), poly-alanine (PA6), and deletions (Bdel1, Bdel2, Bdel3). Dashes represent the deleted amino acids. To highlight the sequence conservation across the Ca_v_2 family, the residues that are not conserved are underlined. Predicted secondary structure (SOPMA algorithm; [43]) is represented by h – for helix, c – for random coil.

The direct coupling model for Ca_v_β regulation predicts that the linker separating the AID from IS6 is a rigid α helix or β sheet. To test this hypothesis, we replaced six consecutive amino acids in the middle of the linker with either glycine (PG6) residues to introduce flexibility, or as a positive control for charge disruptions, with alanines (PA6) to conserve a rigid structure (Fig. 1). Previous circular dichroism studies using peptides designed against wild-type, PG6, and PA6, confirmed the PG6 mutation increased the random coil content from 38.5 to 51%, and the PA6 mutation decreased it to 28% [14]. As a second test of the direct coupling hypothesis, we deleted 1, 2, or 3 amino acids in the middle of the linker in order to alter the orientation of AID-bound β subunit with respect to α_1_. These mutations were introduced into a rat brain α_1_2.2, then studied in HEK-293 cells by whole cell patch clamp electrophysiology.

### β regulation of wild-type α_1_2.2

**Figure 2:**
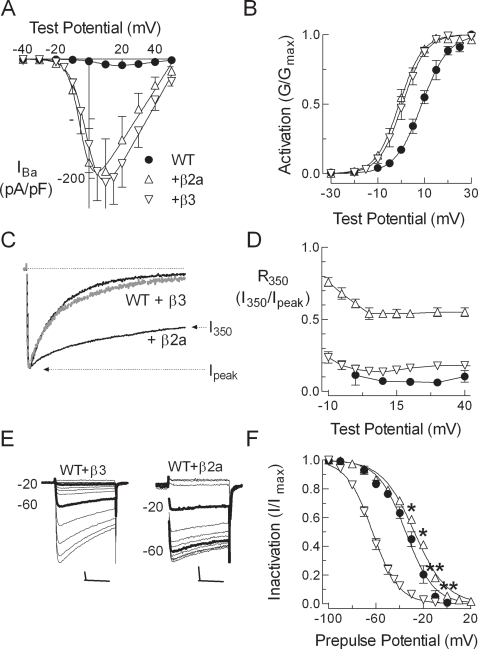
Ca_v_β subunit regulation of α_1_2.2. (A) Average peak currents normalized to cell capacitance. Smooth curves represent fits to the average data using a Boltzmann-Ohm equation. The symbols defined in (A) apply to all panels. (B) Activation represented by the normalized conductance (G/G_max_). (C) Normalized current traces obtained during 350 ms step depolarization to +20 mV from a holding potential of −90 mV. WT+β3 currents are represented by a thick grey line. (D) The residual current at 350 ms of depolarizing pulse was divided by the maximum inward current and plotted against the test potential. (E) Representative current traces obtained during a test pulse to +40 mV after 15 s prepulses to varying potentials from a holding potential of −90 mV. Traces recorded after prepulses to −60 and −20 mV are highlighted to emphasize the β3 induced shift in steady-state inactivation. Scale bar represents 20 ms and 200 pA. (F) Effects of β2a and β3 on steady-state inactivation. The mean normalized amplitude of the currents is expressed as a function of membrane potential and fit with a Boltzmann equation (smooth curves). Data represent mean±SEM, in which the number of cells used to calculate the average is reported in Table 1.

Hallmarks of Ca_v_β regulation of high voltage-activated α_1_ subunits include: their ability to increase the number of functional channels at the plasma membrane (due to both an increase in surface expression of α1 and an increased probability that these channels will open (P_o_) in response to a test depolarization); and to shift the voltage dependence of channel activation [18]. This regulation was reliably detected under our experimental conditions (Fig. 2). Notably, Ca_v_β regulation of inactivation was isoform-specific. Various β2 splice variants, such as β2a, have the ability to dramatically slow inactivation of α_1_2.x currents [19], [20], which can be quantitated by measuring the residual current after 350 ms of depolarization and normalizing it to the peak current amplitude (R_350_; Fig. 2C,D). The ability of β2a to increase R_350_ was relatively voltage independent, therefore the value at +20 mV is representative and is the value reported in the Tables. β3 did not have a statistically significant effect on the R_350_ of WT channels (Fig. 2D, Table 1). Isoform-specific effects on the steady-state inactivation curve (h_∞_) were also observed, with β2a shifting the mid-point (V_50_) +6 mV, while β3 produced a large −30 mV shift in the V_50_ (Fig. 2F). As noted previously, this β3 effect is due to acceleration of closed state inactivation [17]. Preliminary results with β1a and β4 were similar to those obtained with β3 (data not shown), therefore we selected β2a and β3 for further study. In addition, native N-type channels are formed by α_1_2.2 and either β2a or β3 [21], [22]. These electrophysiological signatures provide assays of Ca_v_β regulation that can be used to test α_1_2.2 mutants for loss of regulation (Table 1). Specifically, these βs increase current density over 20-fold and shift the activation curve −10 mV, β2a increase the R_350_ current 10-fold, and β3 shifts steady-state inactivation −34 mV (Table 1).

**Table 1:** Electrophysiological properties of WT, PG6, and PA6 channels and their regulation by β2a and β3.

	Activation		Inactivation		R_350_ or R_25_	Current Density§
	V_50_ (mV)	k	V_50_ (mV)	K		pA/pF
WT	9±1	5.8±0.3 (9)	−35±1	−11.4±0.7 (6)	0.07±0.02 (7)	−10±2 (9)
WT+β2a	−1±1**	4.1±0.2 (16)**	−29±1**	−12.1±0.6 (13)	0.55±0.03 (16)**	−236±57 (16)**
WT+β3	1±1**	4.4±0.2 (7)**	−63±2**	−10.4±0.5 (7)	0.16±0.01 (6)	−248±46 (6)**
PG6	12±2	8.8±1.3 (5)	−29±2	−5.9±1.3 (5)	0.6±0.1 (5)†	−0.9±0.2 (7)
PG6+β2a	12±1	6.6±0.3 (9)	−35±1**	−8.8±0.4 (13)	0.34±0.02 (9)†*	−18±3 (17)**
PG6+β3	11±1	4.1±0.3 (5)**	−41±1**	−6.0±0.9 (7)	0.07±0.07 (4)†**	−3±1 (7)
PA6	14±1	5.9±0.1 (7)	−22±2	−9.2±0.9 (7)	0.10±0.01 (5)	−7±2 (7)
PA6+β2a	6±1**	5.1±0.2 (8)*	−27±1	−10.5±0.5 (8)	0.28±0.02 (8)**	−108±30 (8)**
PA6+β3	0.4±1**	4.9±0.3 (6)**	−50±1**	−10±0.5 (6)	0.034±0.002 (6)*	−298±46 (6)**

The values of V_50_ and k were calculated for each cell, then averaged. R values determined from test pulses to +20 mV. Data shown are mean±SEM from the number of cells shown in parentheses. Statistical significance of the β2a and β3 effects relative to either α_1_ alone (+α_2_δ1) were determined using ANOVA.

† Currents from PG6 were completely inactivated by 350 ms, so the residual current at 25 ms (divided by peak) is reported (R_25_).

§ Current density was estimated from the peak of the *I–V* curve, and statistical significance was determined using Student's *t*-test.

*P<0.05, **P<0.01.

### β regulation of poly-glycine and poly-alanine mutants

**Figure 3:**
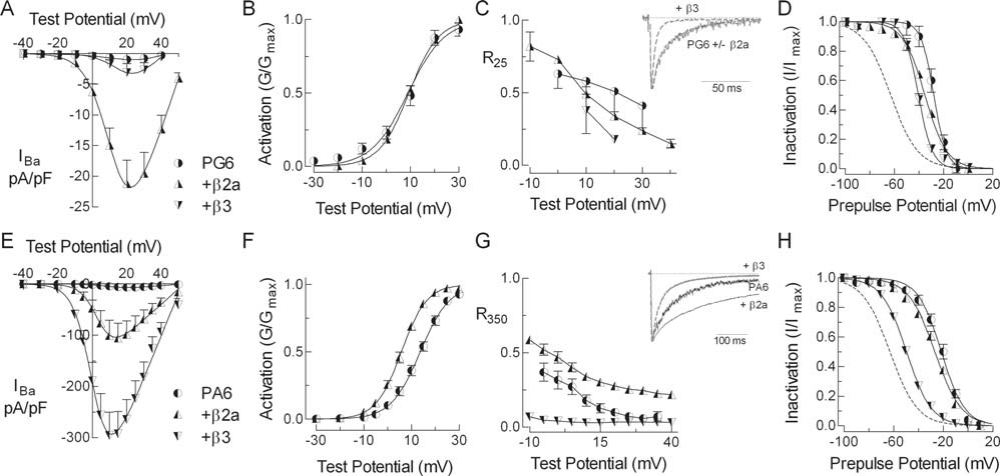
Introduction of the poly-glycine substitution in the α_1_2.2 subunit disrupts (PG6), while poly-alanine substitution (PA6) preserves Ca_v_β regulation. Panels A–D show data obtained with PG6, while panels E–H show data obtained with PA6. (A, E) Peak current-voltage relationships normalized to cell capacitance for the respective α_1_ mutant expressed alone or with β2a or β3. (B, F) Activation represented by the normalized conductance (G/G_max_). The residual current after either 25 ms (C) or 350 ms (G) of depolarization divided by the maximum inward current, and plotted against test potential. Representative traces normalized to the peak inward current are shown in the inset. (D, H) Comparison of the β2a and β3 effects on steady-state inactivation estimated using 15 s prepulses to varying potentials. Dotted lines represent steady-state inactivation measured for WT channels in the presence of β3 (α_1_2.2+α_2_δ1+β3).

Replacement of six amino acids in the IS6-AID linker with glycines (PG6) had a dramatic impact on channel expression and gating (Fig. 3A–C). Current density was decreased 10-fold relative to wild-type (WT) channels and all other notable aspects of β regulation were lost in the PG6 mutant, yet, β2a was still capable of increasing the expression of functional channels (Fig. 3A–D, Table 1). Notably β2a no longer modulated the voltage dependence of activation, β2a no longer increased R_350_, and β3 no longer modulated steady-state inactivation. In contrast to their equipotency at increasing WT currents, β2a increased PG6 currents ∼20-fold, while β3 had no significant effect (Table 1). A second notable difference was the ability of β3 to induce ultra-rapid inactivation of open channels (Fig. 3C). This gain-of-function might be explained by β3 interacting with novel regions of the channel, while the decreased current density is likely due to channels inactivating before opening, a phenomenon that has been observed in Na^+^ and T-type Ca^2+^ channels [23], [24]. In any case, the PG6 mutation largely disrupted normal Ca_v_β regulation as predicted by the direct coupling model.

The IS6-AID linker sequence is more highly conserved than the AID itself. For example, in the α_1_2.x family there is only 1 substitution in 20 residues of the linker (a leucine in α_1_2.1 and α_1_2.2 is substituted by methionine in α_1_2.3), but 3 substitutions in the 20 AID residues. This level of conservation, many of which are charged, suggests that both the structure and properties of amino acid side chains in the linker are important. Therefore, as a control for the PG6 mutation, we attempted to maintain a rigid structure by replacing the same 6 residues with alanine (PA6). As predicted, almost all the hallmarks of β regulation were observed with PA6 channels: βs increased peak currents >15-fold, shifted activation ∼−10 mV, β2a increased R_350_, and β3 shifted the h_∞_ curve (Fig. 3E–H, Table 1). Three aspects of β2a regulation were diminished by the PA6 mutation: one, its ability to increase current density was diminished 2-fold; two, it increased R_350_ to a lesser extent (0.55 in WT vs 0.28 in PA6); and three, its ability to affect steady-state inactivation was lost. In contrast, β3 regulation of PA6 was similar to its regulation of WT channels in terms of current density and ability to shift the h_∞_ curve. The results with PG6 and PA6 are entirely consistent with the IS6-AID linker being a ordered structure as observed in circular dichroism studies of isolated peptides [14].

### β regulation of deletion mutants

**Figure 4:**
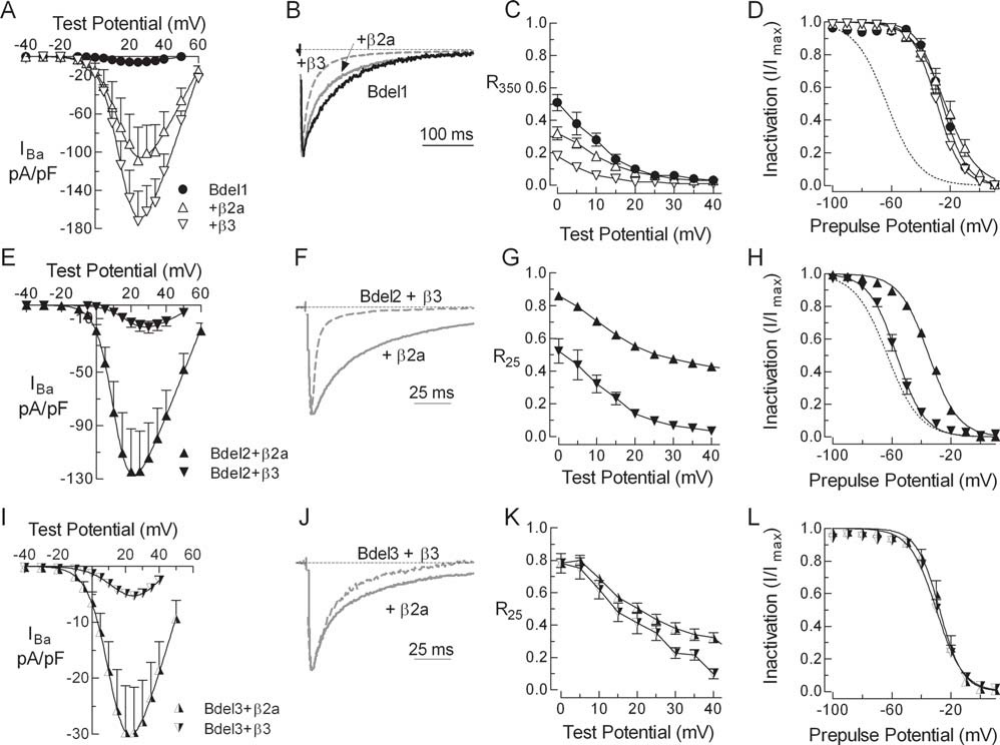
Deletions in the linker between AID and IS6 (Bdels) affect β regulation. Panels A–D show data obtained with Bdel1, panels E–H show data obtained with Bdel2, and panels I–L show data obtained with Bdel3. (A, E, I) Peak current-voltage relationships normalized to cell capacitance for Bdels expressed with β2a or β3. In the absence of a β, only Bdel1 produced detectable currents. (B, F,J) Normalized current traces recorded during depolarizing steps to +20 mV from a holding potential of −90 mV. Residual current after either 350 ms (C) or 25 ms (G, K) of depolarization divided by the maximum inward current, and plotted against test potential. (D, H, L) Comparison of the β2a and β3 effects on steady-state inactivation for Bdels estimated using 15 s prepulses to varying potentials. Dotted lines represent β3 effect on steady-state inactivation for WT channel.

Deletion mutants lacking 1, 2, or 3 amino acids (Bdel1, Bdel2, Bdel3, respectively) in the middle of the IS6-AID linker (see Fig. 1) were prepared using PCR-based mutagenesis. Expression of Bdel1 (with α_2_δ1) led to the appearance of small barium currents whose current density was similar to WT. Coexpression of Bdel1 with either β2a or β3 led to the appearance of robust barium currents, and the stimulation over Bdel1 alone was 13-fold for β2a and 24-fold for β3 (Fig. 4A–D, Table 2). Other than their ability to increase functional channels, most other aspects of β regulation were lost in the Bdel1 mutant: there was no shift in the activation curve, β2a did not affect R_350_, and β3 had no effect on the h_∞_ curve.

**Table 2:** Electrophysiological properties of Bdel1, Bdel2, and Bdel3 channels and their regulation by β2a and β3.

	Activation		Inactivation		R_350_ or R_25_	Current density
	V_50_ (mV)	k	V_50_ (mV)	k		pA/pF
Bdel1	18±1	6.1±0.1 (12)	−25±2	−8.1±0.6 (5)	0.06±0.01 (5)	−7±1 (13)
Bdel1+β2a	17±1	6.2±0.2 (16)	−23±3	−9.1±0.5 (8)	0.04±0.01 (16)	−88±20 (16)**
Bdel1+β3	17±1	5.8±0.2 (10)	−29±1	−8.3±0.2 (9)	0.014±0.002(10)*	−168±29 (10)**
Bdel2+β2a	13±1**	5.8±0.2 (15)	−37±1**	−9.3±0.4 (14)	0.47±0.01 (8)† **	−128±32 (8)**
Bdel2+β3	20±1	5.9±0.3 (7)	−57±2	−8.9±0.8 (7)	0.07±0.02 (6)†	−17±4 (7)
Bdel3+β2a	13±1**	7.1±0.4 (11)	−29±2	−6.3±0.5 (11)	0.38±0.03 (6)† **	−43±11 (8)**
Bdel3+β3	19±2	7.7±0.5 (6)	−30±2	−8.6±0.6 (7)	0.23±0.03 (5)†	−6±1 (9)

Data shown are mean±SEM from the number of cells shown in parentheses. R values were determined from test pulses to +30 mV. Statistical significance for the β effects on Bdel1 were determined with ANOVA. Statistical significance between either Bdel2+β2a and Bdel2+β3, or Bdel3+β2a and Bdel3+β3 were determined by Student's *t*-test.

† Currents from Bdel2 and Bdel3 were completely inactivated by 350 ms, so the R_25_ is reported.

** P<0.01.

Little or no current could be detected from Bdel2 channels when expressed with only α_2_δ1. Measurable currents were detected in 3 of 17 cells, and these currents were too small (current density −0.6±0.2, n = 17) for reliable analysis. No currents could be detected with Bdel3 alone. In contrast, over 300 pA of Ba^2+^ current could be easily measured when a β was cotransfected with these mutants (Fig. 4E). Other signs of β regulation were: one, that Bdel2+β2a currents inactivated 6-fold slower than with β3, but still much faster than WT currents; and two, that β3 modulated the closed-state inactivation of Bdel2 as observed with WT channels, producing a −20 mV shift in the h_∞_ curve (Fig. 4F–H. Similar gain-of-function effects observed with PG6 were also present, with β3 inducing rapid inactivation of currents, and stimulating current density to a lesser extent than β2a. In contrast, inactivation of Bdel3 was not regulated by either β2a or β3, as there was no effect on either open- or closed-state inactivation (Fig. 4J–L).

### Estimation of surface expression and P_o_ of Bdel1

The only typical form of β regulation retained in the Bdel1 mutant was the ability to increase current density. The whole cell current is proportional to the number of channels in the plasma membrane multiplied by their P_o_ (assuming no change in single channel conductance, but see [25]). We hypothesized that trafficking of Bdel1 to the cell surface would be same as for WT [26], since the deletion did not affect the ability of β3 to increase current density. To measure surface expression of Bdel1 and WT channels, we labeled each α_1_ subunit at the N-terminus with GFP, expressed them in HEK-293 cells with α_2_δ1 and β2a, then used confocal microscopy to quantitate GFP signal at the plasma membrane as described previously [27]. Similar amounts of GFP signal were detected at the plasma membrane with Bdel1 (78±7 au/pixels, n = 12) as WT (95±16, n = 8, P = 0.3), and the percent of the GFP signal at the surface was also similar (Bdel1, 41±1; WT, 37±2).

**Figure 5:**
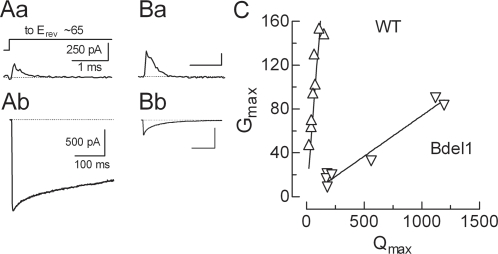
Estimating P_o_ of wild-type and Bdel1 channels. (Aa) Exemplar gating current at reversal potential (∼65 mV) for WT channels expressed with β2a. (Ab) Ionic current trace from the same cell recorded during a depolarizing step to +30 mV from a holding potential of −90 mV. Exemplar gating (Ba) and ionic (Bb) currents for the Bdel1 deletion mutant (also with β2a). Scale bars represent the same units as in panel A. (C) Plot of G_max_ versus Q_max_ where each symbol represents an individual cell. Solid line represents the fit to the data with linear regression. The slope, G/Q, is proportional to maximal channel open probability.

To estimate the effect of the Bdel1 mutation on channel P_o_, we used the method of Yue and coworkers that relies on the ratio of ionic to gating currents [28]. In this method gating currents are measured at the reversal potential and integrated to estimate Q_max_, the whole cell current is normalized to driving force to yield the maximal conductance (G_max_), and the P_o_ is estimated by G_max_/Q_max_. Using the same voltage protocols as Alger et al., (2005) we were able to reliably measure gating currents in cells transfected with β2a, α_2_δ1, and either WT or Bdel1 (Fig. 5). Representative traces from the same cells clearly show that WT channels generate large currents from a small number of channels, while Bdel1 generates smaller currents from a larger number of channels. The slope of the line correlating G_max_ to Q_max_ was 19-fold lower for Bdel1 (0.074±0.004, n = 7) than for WT channels (1.32±0.13, n = 8, P<0.01). This results shows the P_o_ of Bdel1 channels is extremely low, explaining why current density was so low. As noted in the Methods, the transfection protocol was different between WT and Bdel1, thereby precluding direct comparison of G_max_ and Q_max_ in this assay. We conclude that deletion of a single amino acid in the IS6-AID linker abolishes almost all β regulation of the biophysical properties of α_1_2.2, including its ability to increase P_o_, leaving only the β regulation of trafficking.

### Bimolecular Fluorescence Complementation

**Figure 6:**
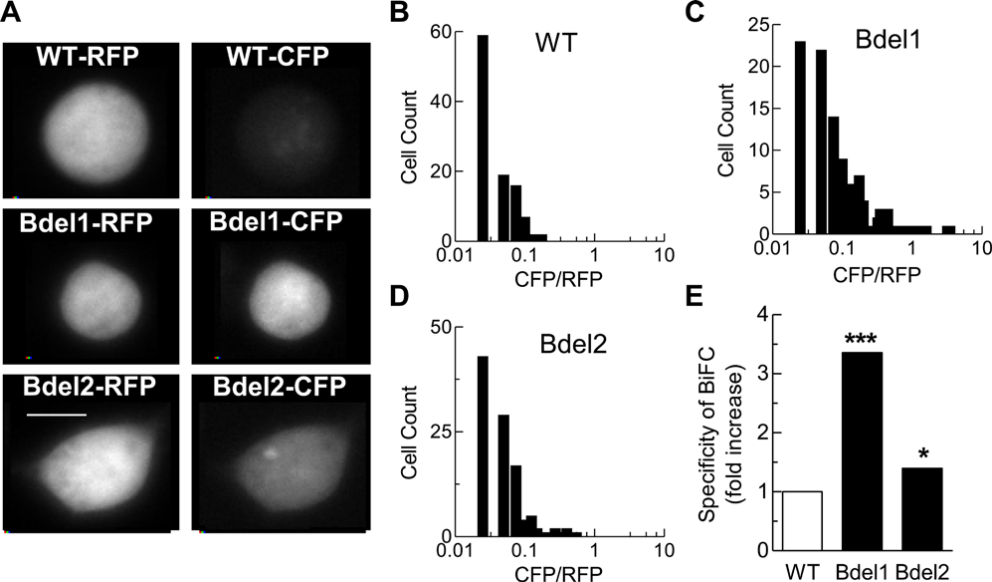
Probing the α_1_-β orientation with bimolecular fluorescence complementation. (A) Images of representative cells transfected with the RFP, mCherry, α_2_δ1, and either WT α_1_2.2, Bdel1, or Bdel2. Transfected cells were identified by their red fluorescence, imaged in the red channel (10 ms), then in the cyan channel (1500 ms). Images were collected with the 40× objective and the spinning disk out (confocal off). Images taken at 100× confirmed that channel proteins were excluded from the nucleus. The images were converted from 24 to 8 bit, and their intensity range was set to 1–260. Inset bar represents 5 µm, and applies to all panels. (B–D) Data used to calculate the specificity of bimolecular fluorescent complementation. Individual cells were imaged in the cyan channel (BiFC signal) and in the red channel (free RFP marker), and the ratio of these signals was calculated. The ratios were then binned using Excel, and plotted using Prism. (B) Results obtained with WT α_1_2.2 tagged with the CFP 1–158 fragment and β3-core tagged with the CFP 159–238 fragment. The median ratio was 0.23. (C) Results obtained with tagged Bdel1, The median ratio was 0.77. (D) Results obtained with tagged Bdel2, The median ratio was 0.32. (E) Specificity of the BiFC signal was determined by dividing the median ratio of the cyan to red fluorescence for the Bdels by the WT median ratio. Results were obtained from the following number of cells: WT, 107; Bdel1, 112; and Bdel2, 111. Asterisks represent statistical significance at the P<0.001 (***) or P<0.5 (*) level.

To address the question of whether the deletions in the IS6-AID linker altered the orientation of β to α_1_2.2 more directly, we utilized bimolecular fluorescence complementation (BiFC) analysis [29]. In this method a fluorescent protein such as CFP is split into two fragments, and then fused to proteins of interest. If the proteins of interest interact, and the two CFP fragments are brought into the proper orientation, then they will reassemble and restore fluorescence. In the present study we relied on the high affinity binding of β to the AID region on α_1_2.2 [30], and fused the fragments of the cyan fluorescent protein (CFP) to the N-terminus of α_1_2.2 and either the N- or C-termini of β3. Preliminary experiments with a full-length β3 tested which combination of tagged proteins could restore the proper orientation (see Methods for all combinations tested), and found the strongest BiFC signal when the big N-terminal fragment of CFP (a.a. 1–158) was fused to the N-terminus of α_1_2.2, and the small C-terminal fragment of CFP was fused to the C-terminus of β3. To restrict the movement of the C-terminal CFP fragment, we truncated the C-terminus of β3 to the same length (β3-core) used in the crystallographic studies [9]. HEK-293 cells were also cotransfected with the RFP, mCherry, which allowed for both selection of transfected cells and calculation of the cyan BiFC to red ratio. As described previously [31], the specificity of the BiFC signal can be calculated from the median ratio of the cyan/red signals (Fig. 6). In our experiments the strongest BiFC signal was observed with tagged Bdel1+β3core constructs, being 3.4-fold higher than WT (Fig. 6). In contrast, the tagged Bdel2+β3core combination produced a lower BiFC signal than Bdel1+β3core, but still higher than WT, a difference that was statistically significant. These results are consistent with the electrophysiology results, where both WT and Bdel2 channels are regulated by β subunits, while Bdel1 is not. We affirm that these results strongly support our hypothesis that the Bdel mutations alter the orientation of the β subunit with respect to the α_1_ subunit.

## Discussion

Since the seminal experiments of Ringer on cardiac muscle contraction [32], it has been recognized that Ca^2+^ entry into cells provides a crucial trigger for life and death processes. Key pathways for Ca^2+^ entry are voltage-gated Ca^2+^ channels, and to fulfill specialized roles these channels have evolved into ten α_1_ subunit genes that are extensively spliced to generate unique channels. Biochemical purification of high voltage-activated channels revealed their multi-subunit structure, being composed of α_1_, α_2_δ, and β subunits [33], [34]. Studies with the cloned subunits clearly established the critical roles of the α_2_δ and β subunits in trafficking and regulating the biophysical properties of the α_1_ subunit. For these reasons, the mechanism of action of these subunits has been extensively investigated. Arguably the greatest progress has been made in understanding the roles and mechanism of action of β subunits [18], [35], [36]. Campbell and coworkers provided a major breakthrough by mapping the site on α_1_ that binds β, termed the AID [7]. The precise details of this interaction were elucidated by X-ray crystallographic studies of AID peptides bound to β2, β3, and β4 [8]–[10]. The AID anchors the β subunit 22 amino acids away from the end of IS6, a distance that is invariant in all HVA α_1_ subunits. S6 segments of Ca^2+^ channels are thought to form an inner gate that opens during channel gating [37], as so clearly observed in K^+^ channel crystal structures [38]. S6 segments are also involved in drug binding, and this binding can be regulated by β subunits [15], [39]. Taken together, these observations led to the direct coupling hypothesis, whereby β modulates α_1_ gating by direct modulation of IS6 movement [8], [15].

In sharp contrast to HVA channels, expression of cloned LVA Ca_v_3 α_1_ subunits produces robust currents with properties that are nearly identical to native T-type currents [40]. In a previous study we exploited this difference to make chimeras that would test the direct coupling hypothesis, moving the I–II loop of α_1_2.2 into α_1_3.1 [14]. Four key results from this study were: one, that some aspects of β regulation could be conferred (β induced a shift in activation curve and slowed open channel inactivation); two, that this regulation was completely dependent on the IS6-AID linker; 3) circular dichroism of peptides corresponding to WT, PA6, and PG6 confirmed the ability of these mutations to disrupt structure; and 4) that this linker was likely to form a rigid structure [14]. Two other interesting conclusions from this previous study were: 1) that the distal portion of the S6 segment is part of an inner gating ring that controls channel inactivation; and 2) that the post-IS6 linker acts as a gating brake in Ca_v_3 channels. The structure of this gating brake is likely an antiparellel helix-loop-helix [41], and is conserved in all three Ca_v_3 channels [42]. A limitation to this previous study was that βs had no effect on P_o_, and β isoform specific regulation of inactivation from open and closed states were not conferred to the chimera. Therefore, in the present study we have explored the importance of the IS6-AID structure by mutating this region in α_1_2.2.

We show that mutations that induce flexibility and destabilize α-helices and β-sheets, as with PG6, totally abolish β subunit regulation. Specifically, β subunits no longer shift the activation curve, β3 no longer shifts the h_∞_ curve, and β2a no longer increases plateau currents. As a control, we show that replacement of these same amino acids with alanines largely retain β regulation of gating. Interestingly, β3 accelerated inactivation kinetics of PA6, and β2a's ability to affect inactivation was altered. Two possible explanations for the loss of β regulation of PA6 are: one, particular amino acid residues may play a role (e.g. charged residues stabilize interactions with other channel regions) or two, the structure of PA6 is not identical to WT.

**Figure 7:**
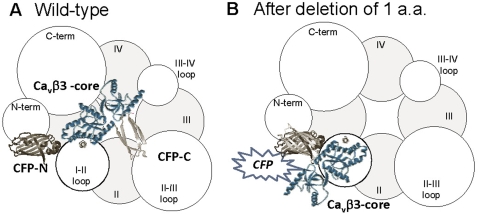
Model showing possible orientations of β with respect to α_1_ assuming a β sheet structure at the site of deletion. (A) Model showing the orientation of β-subunit in wild-type, and (B) after deletion of 1 amino acid. The β3 core structure was modeled from PDB code 1VYT [9]. The CFP, Cirulean, was modeled from PDB code 2QYT [57]. The fragments of CFP were generated using PyMOLWin (Delano Scientific), where CFP-N corresponds to residues 1–158, and CFP-C corresponds to residues 159–238. The approximate size of the α_1_2.2 domains and linkers were estimated using the method of Helton and Horne, where the volume occupied by each segment is calculated from the number of amino acids in each segment [45]. The β3 subunit was scaled using the same method.

As a second test of the direct coupling hypothesis, we deleted 1, 2, or 3 amino acids from the middle of the IS6-AID linker. The key results were loss of β regulation in Bdel1 and Bdel3, and retention of regulation in Bdel2. A limitation to these studies is that little or no currents could be recorded from Bdel2 or Bdel3 when expressed alone (+α_2_δ1), so the presence or absence of β regulation could only be inferred by comparisons between β2a and β3. Nevertheless, the ability of βs to increase functional channels at the plasma membrane was retained in all 3 Bdel mutants. We conclude that the central region of the IS6-AID adopts a β-sheet structure. Deletion of 1 or 3 amino acids in this β-sheet would alter the orientation of Ca_v_β with respect to α1 by 180°, while deletion of two would return the orientation back to WT (Fig. 7). Although β subunits were crystallized with peptides corresponding to the AID sequence, these peptides did not include the 20 amino acids of the IS6-AID linker, consequently the exact structure of this region was not determined. Opatowsky et al., (2004) proposed an interesting hypothesis whereby β binds to a disordered AID and induces its folding into an α helix that extends all the way to IS6 [10]. Secondary structure prediction programs [43] suggest that this linker has a high tendency to form an α helix, and near the middle of the linker an equal tendency to form a β sheet. Deletions in this region may preferentially stabilize the β-sheet structure. Regardless of the structure, the orientation of β subunit is critical for its ability to regulate the biophysical properties of α_1_2.2 channels. We hypothesize that β is precisely anchored at the AID to orient other parts of β towards regions of α_1_ that control channel opening (thereby modulating P_o_ and the activation curve) and channel inactivation.

The palmitic moieties of β2a may alter this orientation, thereby altering its interaction with inactivation gates [19]. This difference in orientation is supported by the finding that the ability of β2a and β3 to increase current density and their effect on inactivation kinetics varied between the mutants.

Mutations in the AID that weaken its interaction with β subunits may allow a new orientation, which explains why the W391A mutation in α_1_2.2 abolishes β1 but not β2a regulation [30]. Alternative splicing of either β subunits [44]–[46] or α_1_ subunits may provide additional isoform specific regulation [47]. The crystal structures of βs provided clues for additional binding sites: one, the SH3 domain, which is a common structural motif at protein interaction sites, and two, the large groove between the GK and SH3 domain [8]–[10]. Notably, a rigid IS6-AID linker would orient this groove directly below the cytoplasmic face of α_1_. The other interaction sites on α_1_ are likely to be of much lower affinity, allowing regulatory proteins such as G protein βγ to disrupt these interactions. In this scenario, Gβγ could shift channels into reluctant states without complete dissociation of Ca_v_β from the AID, which reconciles conflicting observations [6], [8], [48]. Mutational studies support the hypothesis that all voltage-gated channels have a similar inverted teepee structure where the intracellular gate is formed by a bundle crossing of S6 segments as observed in the crystal structure of Shaker K^+^ channels [15], [37], [38]. The precise positioning of Ca_v_β near this bundle crossing would allow it to interact with any of the post-S6 segments. Inappropriate interactions appear to severely accelerate channel inactivation, as observed with the needle-like kinetics of PG6 and Bdel2 induced by β3, which may be due to novel interactions with other post-S6 segments. Accordingly, De Waard and colleagues have provided evidence that post-IIIS6, as well as the carboxyl terminus, play important roles in Ca_v_β regulation of α_1_2.1 [49], [50]. An alternative version of the direct coupling hypothesis is that the other α_1_-β interactions serve mainly as a fulcrum, allowing β to restrain movement of IS6. The gating current studies show that deletion of one amino acid from IS6-AID linker largely disrupts βs ability to increase P_o_, therefore, we conclude that orientation of β is critical for its regulation of the biophysical properties of α_1_2.2, and confirm the importance of additional points of contact between the two subunits.

## Materials and Methods

### Site-directed mutagenesis

The starting material (wild-type, WT) for these studies was the cDNA encoding the “a” isoform of rat brain α_1_2.2 cDNA (GenBank entry #AF055477) cloned into the plasmid vector pcDNA6 [51]. A 1.5 kb fragment was subcloned into pCR2.1-TOPO (Invitrogen, Carlsbad, CA), then mutated using the Quikchange® protocol and *Pfu* Ultra DNA polymerase (Stratagene, La Jolla, CA). Oligonucleotide primers were obtained from Invitrogen and used without purification. All restriction enzymes were purchased from New England Biolabs (Ipswich, MA). The full-length cDNA was reassembled in the original plasmid vector that was cut with *Asc*I and *Bsi*WI by ligating the following fragments: *Asc*I(32)/*Blp*I(355), *Blp*I/*Sac*I (1407), and *Sac*I/*Bsi*WI (2991). The mutations were contained in the *Blp*I/*Sac*I fragment, and the sequence of this fragment was verified for each mutant by automated sequencing at the University of Virginia Biomolecular Research Facility.

GFP-tagged versions of α_1_2.2 were made by PCR amplifying a 1.5 kb fragment corresponding to the 5′ end. The 5′ primer included a *Bgl*II site to allow in-frame cloning with GFP in the vector pAcGFP-C1 (Clontech). The full-length clone was made by ligating the following fragments: *Bgl*II (5′ UTR)/*Sac*I (1407), *Sac*I/*Sph*I(4937), and *Sph*I/*Bam*HI (7345)

### Transfections

293 cells (human embryonic kidney, #CRL-1573, American Type Culture Collection, Manassas, VA) were grown in Dulbecco's modified medium F12 (Invitrogen) supplemented with 10% fetal calf serum, pencillin G (100 units/ml), and streptomycin (0.1 mg/ml). Cells were transiently transfected using JET-PEI (Polyplus, Illkirch, France) and 2 µg of plasmid DNAs encoding WT or mutant α_1_2.2, 1 µg rabbit α_2_δ1 subcloned into pcDNA3.1, 0.25 µg green fluorescent protein (pGreen Lantern, Invitrogen), and in the absence or presence of 1 µg Ca_v_β subunit: either rat or human β2a [46], and rat β3 [52]. Cells were split 24 h after transfection, seeded on poly-lysine coated cover-slips, and maintained in normal growth media at 37°C. Recordings were made 2–8 hours later, or after overnight incubation at 29°C [53].

Transfection of α_1_2.2, and any β under these conditions led to the appearance of robust currents that could be reliably clamped. In contrast, transfection of α1 alone produced little or no currents. Addition of α_2_δ1 increased the amplitude of the currents 2- to 5-fold, and increased the percentage of cells with current. With some of the mutants (e.g. Bdel2, Bdel3), there was no detectable current under these conditions. To boost expression, transfections were modified as follows: 1) increasing the amount of mutant α_1_2.2 and α_2_δ1 plasmids to 3 µg, 2) adding 0.5 µg of plasmid containing the SV40 T antigen, and 3) incubating the cells 48 hours before plating onto chips. Under these conditions currents from all the mutants could be reliably measured, however, wild-type currents were so large that in many cases they saturated the amplifier (>20 nA). As a consequence, the ability of β subunits to stimulate expression of functional channels is underestimated. Similar experimental conditions have been used in previous studies to record recombinant Ca_v_2.2 currents [28], [54].

### Electrophysiology

Electrophysiological experiments were carried out using the whole cell configuration of the patch clamp technique. Recordings were obtained using an Axopatch 200A amplifier equipped with a CV201A headstage. The amplifier was connected to a computer (Dell, Round Rock, TX) through a Digidata 1200 A/D converter, and controlled using pCLAMP 9.2 software (Axon Instruments, Union City, CA). Currents were recorded using the following external solution (in mM): 10 BaCl_2_, 156 tetraethyl ammonium (TEA) chloride, and 10 HEPES, pH adjusted to 7.4 with TEA-OH. The internal pipette solution contained the following (in mM): 125 CsCl, 10 BAPTA, 2 CaCl_2_, 1 MgCl_2_, 4 Mg-ATP, 0.3 Li-GDPβS, and 10 HEPES, pH adjusted to 7.2 with CsOH.

Pipettes were made from TW-150-3 capillary tubing (World Precision Instruments, Inc., Sarasota, FL). Initial pipette resistance was typically 2–3 MΩ. Access resistance and cell capacitance were measured using on-line exponential fits to a capacitance transient (Membrane Test, Clampex). Cell capacitance ranged between 8–30 pF. Access resistance averaged 4.2 MΩ. Data from cells where the access resistance exceeded 5.5 MΩ were discarded. Series resistance was compensated between protocols to 70% (prediction and correction; 10 µs lag), resulting in maximal residual voltage error below 1.6 mV during measurement of the current-voltage relationship. Data were collected at room temperature.

To balance the effects of inactivation, slow recovery, and rundown, we selected 350 ms pulses for the current-voltage protocol, and an inter-sweep interval of 20 s. Peak currents at each voltage step were used to calculate the voltage dependence of activation (V_0.5_, and *k*), and the conductance (*G*) as described previously [27]. The current at the end of the depolarizing pulse was also measured, and divided by the peak current in that pulse to derive the R_350_ value. The voltage dependence of steady-state inactivation was estimated using 15 s prepulses to varying potentials followed by a test pulse to +40 mV to measure channel availability (*h*). The current elicited during each test pulse was normalized to that observed when the holding potential was −90 mV (*I/Imax*), and the data from each cell were fit with a Boltzmann equation using Prism® software (version 5, Graphpad Software, San Diego, CA). Results are presented as mean±SEM. Significant differences in the average data were analyzed using one-way ANOVA followed by Bonferroni's multiple comparison test (GraphPad Prism).

### Confocal microscopy

Images of live cells were collected using a Cooke Sensicam QE CCD camera (Romulus, MI) mounted on an Olympus BX61WI microscope equipped with an Olympus confocal spinning disk unit (Melville, NY). Channel localization was visualized by measuring the green fluorescent signal from GFP fused to the N-termini of either WT or Bdel1. Data were acquired under identical conditions, and then analyzed using IPLab 4.0 (Scanalytics, Fairfax, VA) as described previously [27]. Plasma membranes were labeled with FM 4-64 (Invitrogen) following the supplier's recommendations. Live cells were treated for at least 5 min at 4°C, and then imaged at 4°C. The FM-4-64 signal was used to localize the plasma membrane, and the amount of green fluorescent signal (arbitrary units, au) was measured and normalized to the number of pixels in this area. For both channels the GFP signal was evenly distributed in the plasma membrane.

### Bimolecular Fluorescence Complementation (BiFC)

In preliminary experiments we fused either the CFP N-terminal fragment (a.a. 1–158, abbreviated B) or C-terminal fragment (a.a. 159–238, abbreviated S) to the amino terminus of WT α_1_2.2 (a1B–B and a1B–S); and fused both CFP fragments to either the N- or C-termini of rat β3 (SNB3, BNB3, SCB3, BCB3). The CFP fragments and β3 coding region were PCR amplified with primers that added unique restriction sites in the correct reading frame. The CFP fragments were ligated to full-length α_1_2.2 cDNA using *Kpn*I (polylinker) and *Asc*I (−63), thereby creating a flexible 21 a.a. linker (5 glycines) from the 5′ untranslated region. The CFP-β3 linker included an *Sbf*I site that encoded PAGAT, while the β3-CFP linker included a *Bsp*EI site that encoded SGAT. Of the four combinations that could produce a BiFC signal, the largest signal was detected with a1B–B+SCB3. The crystal structure of β3 was obtained with a construct called β3core (B3c). In agreement with previous reports [9], [55], we found that B3c remains functional despite having the non-conserved amino and carboxy termini removed (results not shown). Since the 121 a.a. of the C-terminus might allow flexibility that would obscure the orientation of β, we prepared a C-terminal CFP (a.a. 159–238; SCB3c) fused to the β3core. The Bdel1-B and Bdel2-B were prepared using the same *Kpn*I/*Asc*I cloning strategy as for WT. These constructs (88 ng each) were transiently transfected into HEK-293 cells along with α_2_δ1 and the red fluorescent protein (RFP), mCherry [56]. Similar results were obtained using the RFP, DsRed-Monomer (Clontech), so the results were pooled. After 18 hrs the cells were plated onto polylysine-treated glass bottom dishes (Fluorodish, World Precision Instruments, Saratoga, FL). Transfected cells were identified by their red fluorescence, and their red and cyan fluorescence signals were collected with IPLab software and the Olympus microscope described above (40× objective, 2×2 binning, confocal off). Data were background subtracted using a region devoid of cells, and the ratio of green to red signal for each cell was calculated. Following the method of Shyu et al. [31], BiFC specificity was determined using the median of the CFP/RFP signal for each condition. Statistical analysis was performed using one-way ANOVA with the Kruskal-Wallis post test using GraphPad Prism.

## References

[1] Berridge MJ, Bootman MD, Lipp P (1998). Calcium–a life and death signal.. Nature.

[2] Lacerda AE, Kim HS, Ruth P, Perez-Reyes E, Flockerzi V (1991). Normalization of current kinetics by interaction between the α_1_ and β subunits of the skeletal muscle dihydropyridine-sensitive Ca^2+^ channel.. Nature.

[3] Mori Y, Friedrich T, Kim MS, Mikami A, Nakai J (1991). Primary structure and functional expression from complementary DNA of a brain calcium channel.. Nature.

[4] Singer D, Biel M, Lotan I, Flockerzi V, Hofmann F (1991). The roles of the subunits in the function of the calcium channel.. Science.

[5] Dolphin AC, Wyatt CN, Richards J, Beattie RE, Craig P (1999). The effect of α_2_δ and other accessory subunits on expression and properties of the calcium channel α1G.. J Physiol.

[6] Richards MW, Butcher AJ, Dolphin AC (2004). Ca^2+^ channel β-subunits: structural insights AID our understanding.. Trends Pharmacol Sci.

[7] Pragnell M, De Waard M, Mori Y, Tanabe T, Snutch TP (1994). Calcium channel β-subunit binds to a conserved motif in the I–II cytoplasmic linker of the α_1_-subunit.. Nature.

[8] Van Petegem F, Clark KA, Chatelain FC, Minor DL (2004). Structure of a complex between a voltage-gated calcium channel β-subunit and an α-subunit domain.. Nature.

[9] Chen Y-H, Li M-H, Zhang Y, He L-l, Yamada Y (2004). Structural basis of the α1-β subunit interaction of voltage-gated Ca^2+^ channels.. Nature.

[10] Opatowsky Y, Chen CC, Campbell KP, Hirsch JA (2004). Structural analysis of the voltage-dependent calcium channel β subunit functional core and its complex with the α1 interaction domain.. Neuron.

[11] Hanlon MR, Berrow NS, Dolphin AC, Wallace BA (1999). Modelling of a voltage-dependent Ca^2+^ channel β subunit as a basis for understanding its functional properties.. FEBS Lett.

[12] Harry JB, Kobrinsky E, Abernethy DR, Soldatov NM (2004). New short splice variants of the human cardiac Ca_v_β2 subunit: redefining the major functional motifs implemented in modulation of the Ca_v_1.2 channel.. J Biol Chem.

[13] Cohen RM, Foell JD, Balijepalli RC, Shah V, Hell JW (2005). Unique modulation of L-type Ca2+ channels by short auxiliary β1d subunit present in cardiac muscle.. Am J Physiol Heart Circ Physiol.

[14] Arias JM, Murbartián J, Vitko I, Lee JH, Perez-Reyes E (2005). Transfer of β subunit regulation from high to low voltage-gated Ca^2+^ channels.. FEBS Letters.

[15] Hering S (2002). β-subunits: fine tuning of Ca^2+^ channel block.. Trends Pharmacol Sci.

[16] Wakamori M, Mikala G, Mori Y (1999). Auxiliary subunits operate as a molecular switch in determining gating behaviour of the unitary N-type Ca^2+^ channel current in Xenopus oocytes.. J Physiol.

[17] Yasuda T, Lewis RJ, Adams DJ (2004). Overexpressed Ca_v_β3 inhibits N-type (Ca_v_2.2) calcium channel currents through a hyperpolarizing shift of ultra-slow and closed-state inactivation.. J Gen Physiol.

[18] Dolphin AC (2003). Beta subunits of voltage-gated calcium channels.. J Bioenerg Biomembr.

[19] Qin N, Platano D, Olcese R, Costantin JL, Stefani E (1998). Unique regulatory properties of the type 2a Ca^2+^ channel β subunit caused by palmitoylation.. Proceedings of the National Academy of Sciences of the United States of America.

[20] Sokolov S, Weiss RG, Timin EN, Hering S (2000). Modulation of slow inactivation in class A Ca^2+^ channels by β-subunits.. J Physiol.

[21] Vance CL, Begg CM, Lee WL, Haase H, Copeland TD (1998). Differential expression and association of calcium channel α_1B_ and β subunits during rat brain ontogeny.. J Biol Chem.

[22] Scott VE, De Waard M, Liu H, Gurnett CA, Venzke DP (1996). β subunit heterogeneity in N-type Ca^2+^ channels.. Journal of Biological Chemistry.

[23] Gonoi T, Hille B (1987). Gating of Na channels. Inactivation modifiers discriminate among models.. J Gen Physiol.

[24] Frazier CJ, Serrano JR, George EG, Yu X, Viswanathan A (2001). Gating kinetics of the α1I T-type calcium channel.. J Gen Physiol.

[25] Schjott JM, Hsu SC, Plummer MR (2003). The neuronal β4 subunit increases the unitary conductance of L-type voltage-gated calcium channels in PC12 cells.. J Biol Chem.

[26] Brust PF, Simerson S, Mccue AF, Deal CR, Schoonmaker S (1993). Human neuronal voltage-dependent calcium channels: studies on subunit structure and role in channel assembly.. Neuropharmacology.

[27] Vitko I, Bidaud I, Arias JM, Mezghrani A, Lory P (2007). The I–II loop controls plasma membrane expression and gating of Ca_v_3.2 T-type Ca^2+^ channels: a paradigm for Childhood Absence Epilepsy.. Journal of Neuroscience.

[28] Agler HL, Evans J, Tay LH, Anderson MJ, Colecraft HM (2005). G protein-gated inhibitory module of N-type (Ca_v_2.2) Ca^2+^ channels.. Neuron.

[29] Kerppola TK (2006). Design and implementation of bimolecular fluorescence complementation (BiFC) assays for the visualization of protein interactions in living cells.. Nat Protoc.

[30] Leroy J, Richards MS, Butcher AJ, Nieto-Rostro M, Pratt WS (2005). Interaction via a key tryptophan in the I–II linker of N-type calcium channels is required for β1 but not for palmitoylated β2, implicating an additional binding site in the regulation of channel voltage-dependent properties.. J Neurosci.

[31] Shyu YJ, Liu H, Deng X, Hu CD (2006). Identification of new fluorescent protein fragments for bimolecular fluorescence complementation analysis under physiological conditions.. Biotechniques.

[32] Ringer S (1883). A further contribution regarding the influence of the different constituents of the blood on the contraction of the heart.. J Physiol.

[33] Wolf M, Eberhart A, Glossmann H, Striessnig J, Grigorieff N (2003). Visualization of the domain structure of an L-type Ca^2+^ channel using electron cryo-microscopy.. J Mol Biol.

[34] Leung AT, Imagawa T, Block B, Franzini-Armstrong C, Campbell KP (1988). Biochemical and ultrastructural characterization of the 1,4-dihydropyridine receptor from rabbit skeletal muscle. Evidence for a 52,000 Da subunit.. Journal of Biological Chemistry.

[35] Birnbaumer L, Qin N, Olcese R, Tareilus E, Platano D (1998). Structures and functions of calcium channels β subunits.. Journal of Bioenergetics and Biomembranes.

[36] Arikkath J, Campbell KP (2003). Auxiliary subunits: essential components of the voltage-gated calcium channel complex.. Curr Opin Neurobiol.

[37] Soldatov NM (2003). Ca^2+^ channel moving tail: link between Ca^2+^-induced inactivation and Ca^2+^ signal transduction.. Trends Pharmacol Sci.

[38] Long SB, Campbell EB, Mackinnon R (2005). Crystal structure of a mammalian voltage-dependent Shaker family K^+^ channel.. Science.

[39] Striessnig J, Grabner M, Mitterdorfer J, Hering S, Sinnegger MJ (1998). Structural basis of drug binding to L Ca^2+^ channels.. Trends Pharmacol Sci.

[40] Perez-Reyes E (2003). Molecular physiology of low-voltage-activated T-type calcium channels.. Physiol Rev.

[41] Arias-Olguín II, Vitko I, Fortuna M, Baumgart JP, Sokolova S (2008). Characterization of the gating brake in the I–II loop of Ca_v_3.2 T-type Ca^2+^ channels.. J Biol Chem.

[42] Baumgart JP, Vitko I, Bidaud I, Kondratskyi A, Lory P (2008). I–II loop structural determinants in the gating and surface expression of low voltage-activated calcium channels.. PLoS ONE.

[43] Geourjon C, Deleage G (1995). SOPMA: significant improvements in protein secondary structure prediction by consensus prediction from multiple alignments.. Comput Appl Biosci.

[44] Qin N, Olcese R, Zhou J, Cabello OA, Birnbaumer L (1996). Identification of a second region of the β-subunit involved in regulation of calcium channel inactivation.. American Journal of Physiology.

[45] Helton TD, Horne WA (2002). Alternative splicing of the β4 subunit has α_1_ subunit subtype-specific effects on Ca^2+^ channel gating.. J Neurosci.

[46] Takahashi SX, Mittman S, Colecraft HM (2003). Distinctive modulatory effects of five human auxiliary β2 subunit splice variants on L-type calcium channel gating.. Biophys J.

[47] Sandoz G, Bichet D, Cornet V, Mori Y, Felix R (2001). Distinct properties and differential β subunit regulation of two C-terminal isoforms of the P/Q-type Ca^2+^-channel α_1A_ subunit.. Eur J Neurosci.

[48] Sandoz G, Lopez-Gonzalez I, Grunwald D, Bichet D, Altafaj X (2004). Ca_v_β-subunit displacement is a key step to induce the reluctant state of P/Q calcium channels by direct G protein regulation.. Proc Natl Acad Sci U S A.

[49] Geib S, Sandoz G, Cornet V, Mabrouk K, Fund-Saunier O (2002). The interaction between the I–II loop and the III–IV loop of Ca_v_2.1 contributes to voltage-dependent inactivation in a β-dependent manner.. J Biol Chem.

[50] Walker D, Bichet D, Campbell KP, De Waard M (1998). A β4 isoform-specific interaction site in the carboxyl-terminal region of the voltage-dependent Ca^2+^ channel α1A subunit.. Journal of Biological Chemistry.

[51] Lin Z, Haus S, Edgerton J, Lipscombe D (1997). Identification of functionally distinct isoforms of the N-type Ca^2+^ channel in rat sympathetic ganglia and brain.. Neuron.

[52] Castellano A, Wei XY, Birnbaumer L, Perez-Reyes E (1993). Cloning and expression of a third calcium channel β subunit.. Journal of Biological Chemistry.

[53] Feng Z-P, Arnot MI, Doering CJ, Zamponi GW (2001). Calcium channel β subunits differentially regulate the inhibition of N-type channels by individual Gβ isoforms.. J Biol Chem.

[54] Stephens GJ, Page KM, Bogdanov Y, Dolphin AC (2000). The α1B Ca^2+^ channel amino terminus contributes determinants for β subunit-mediated voltage-dependent inactivation properties.. J Physiol.

[55] He LL, Zhang Y, Chen YH, Yamada Y, Yang J (2007). Functional modularity of the β-subunit of voltage-gated Ca^2+^ channels.. Biophys J.

[56] Shaner NC, Campbell RE, Steinbach PA, Giepmans BN, Palmer AE (2004). Improved monomeric red, orange and yellow fluorescent proteins derived from Discosoma sp. red fluorescent protein.. Nat Biotechnol.

[57] Malo GD, Pouwels LJ, Wang M, Weichsel A, Montfort WR (2007). X-ray structure of cerulean GFP: a tryptophan-based chromophore useful for fluorescence lifetime imaging.. Biochemistry.

